# Simulation of a Steep-Slope p- and n-Type HfS_2_/MoTe_2_ Field-Effect Transistor with the Hybrid Transport Mechanism

**DOI:** 10.3390/nano13040649

**Published:** 2023-02-07

**Authors:** Juan Lyu, Jian Gong

**Affiliations:** School of Physical Science and Technology, Inner Mongolia University, Hohhot 010021, China

**Keywords:** cold source, subthreshold swing

## Abstract

The use of a two-dimensional (2D) van der Waals (vdW) metal-semiconductor (MS) heterojunction as an efficient cold source (CS) has recently been proposed as a promising approach in the development of steep-slope field-effect transistors (FETs). In addition to the selection of source materials with linearly decreasing density-of-states-energy relations (D(E)s), in this study, we further verified, by means of a computer simulation, that a 2D semiconductor-semiconductor combination could also be used as an efficient CS. As a test case, a HfS_2_/MoTe_2_ FET was studied. It was found that MoTe_2_ can be spontaneously p-type-doped by interfacing with n-doped HfS_2_, resulting in a truncated decaying hot-carrier density with an increasing p-type channel barrier. Compared to the conventional MoTe_2_ FET, the subthreshold swing (SS) of the HfS_2_/MoTe_2_ FET can be significantly reduced to below 60 mV/decade, and the on-state current can be greatly enhanced by more than two orders of magnitude. It was found that there exists a hybrid transport mechanism involving the cold injection and the tunneling effect in such a p- and n-type HfS_2_/MoTe_2_ FET, which provides a new design insight into future low-power and high-performance 2D electronics from a physical point of view.

## 1. Introduction

As the scaling of silicon transistors is approaching its physical limits, in accordance with Moore’s Law (scaling law), the principle that has governed the information-technology revolution since the 1960s, performance improvements in integrated circuits are being delivered at a slower pace. Regarding today’s sub-10 nm technology nodes, scaling is increasingly challenging because the power densities of chips increase significantly and the gate electrostatics of devices are severely degraded [1]. In a conventional field-effect transistor (FET), the subthreshold swing (SS) is limited by the thermionic emission above the channel barrier, so that the lower bound (Boltzmann limit) of SS is 60 mV/decade at room temperature [2,3]. Appropriate semiconductor materials with tunable electronic properties are crucial to the fabrication of low-power transistors with steep-slope SSs. Two-dimensional (2D) semiconductor materials, such as transition-metal dichalcogenides (TMDs), are considered excellent channel-material candidates for electronic devices in the post-silicon era [4,5,6]. Two-dimensional materials can form vertical heterostructures through unique van der Waals (vdW) interactions without Fermi level (FL) pinning effects, providing a promising solution to overcome the poor contact quality of the metal–semiconductor (MS) interface in 2D field-effect transistors (FETs) [7,8,9,10,11,12].

Our previous calculations confirmed that 2D vdW MS interfaces with the desired density-of-states-energy relations (DOS(E)s) are the general ingredients for steep-slope cold-source EFTs (CS-FETs) [13]. In a CS-FET, the hot-carrier (HC) density of states (DOS) of the CS should decrease with an increasing channel barrier. The function of the CS can be understood by referring to the Landauer-Büttiker formula:

(1) $$I=\frac{2e}{h}\int_{-\infty }^{+\infty }T(E)D(E)\;\left[f\left(E-E_{F}\left(S\right)\right)-f\left(E-E_{F}\left(D\right)\right)\right]\;dE$$

where T (E) is the transmission probability, D (E) is the DOS, f (E) is the Boltzmann distribution function, and E_F_ (S) and E_F_ (D) are the Fermi levels of the source and drain electrode, respectively. In a conventional n-type FET, in the on state, electrons are injected from the highly n-type-doped (degenerate) semiconducting region, the conduction band DOS of which is an increasing function of energy, or from normal metal, the DOS of which is essentially independent of energy. In the off state, due to the thermal Boltzmann distribution, electrons in the source have an energy distribution (n (E) = D (E)*f (E)) that spreads (thermal tail) to a value exceeding the potential barrier (hot electrons) [14]. Due to the non-decreasing D (E) relationship, the hot-electron density can increase with energy, which sets a 60 mV/decade limit on SS. However, if injection is from a material whose HC DOS decreases with an increasing channel barrier, a super-exponentially decreasing HC density n (E) can be achieved, leading to more localized carrier distributions around the FL without a long thermal tail above the channel barrier. As a result, the device can be switched off faster because the thermal tail can be more effectively cut off by D (E), according to the above formula, thus breaking the SS limit of conventional FETs. Here, a natural question arises: is it possible to use a doped 2D semiconductor with a band gap below the Fermi level as a more effective CS to further lower the SS? With regard to the above question, we investigated the feasibility of using a 2D semiconductor-semiconductor combination as an efficient CS for a steep-slope FET in this article. MoTe_2_ is an attractive semiconductor channel material with bipolar carrier-transport characteristics [15,16,17,18,19,20,21,22,23]. Hence, a monolayer MoTe_2_-based high-mobility FET with an n-doped 2D HfS_2_ source was studied as a test case. By analyzing the D (E) relation, n-doped HfS_2_ CS was found to result in SSs below 60 mV/decade, which can be explained by its desired hot-carrier distributions n (E) and the gate-tunable source-channel barrier heights. This work opens up new opportunities at the confluence of 2D semiconductors and low-power electronics.

## 2. Computational Methods

The electronic properties and transport-simulation calculations were carried out using the first-principle software package Atomistix ToolKit (ATK) [24,25], based on density functional theory (DFT) in combination with the nonequilibrium Green’s function (NEGF). The exchange-correlation energies were processed according to generalized gradient approximation (GGA) in the form of the Perdew–Burke–Ernzerhof (PBE) functional [26]. The double-zeta plus polarization (DZP) basis set was employed. Geometry optimization was performed based on the periodic supercell method. The k-point mesh was sampled at 1 × 7 × 9 for the calculation of structure-relaxation and electronic properties, and the grid cutoff energy was set at 85 Hartrees. A vacuum region of 18 Å was used to avoid spurious interaction between periodic images. The atomic positions were fully relaxed until the maximum energy difference and residual forces converged to 10^−5^ eV and 0.05 eV/Å. Van der Waals correction was performed according to the Grimme DFT-D2 method. The calculation of the carrier mobility of MoTe_2_ was performed using the Vienna ab initio simulation package with the same parameter settings [27,28,29,30,31,32,33], which were based on deformation potential theory [34,35].

Quantum transport simulation was performed according to DFT coupled with the NEGF method. The k-point grids used to calculate the transport characteristics were set at 1 × 11 × 133, and the temperature was set at 300 K. The cutoff of the real-space mesh was 150 Rydbergs. The drain current at a given gate voltage *V*_G_ and bias voltage *V*_SD_ was calculated using the Landauer-Büttiker formula [36]. The transmission coefficient *T* (E) is the k-dependent transmission coefficient $T^{k_{\|}}$ (E) average over the two-dimensional Brillouin zone perpendicular to the transport direction. The reciprocal lattice vector $k_{∥}$ is vertical to the transport direction. The k-dependent transmission coefficient at energy E is as follows:

(2) $$T^{k_{∥}}\left(E\right)=\mathrm{Tr}\left\{\Gamma_{L}^{k_{∥}}\left(E\right)G^{k_{∥}}\left(E\right)\Gamma_{R}^{k_{∥}}\left(E\right){\left[G^{k_{∥}}\left(E\right)\right]}^{†}\right\}$$

where $G^{k_{∥}}\left(E\right)$ and $\left\{{\left[G^{k_{∥}}\left(E\right)\right]}^{†}\right\}$ represent the retarded (advanced) Green function and $\Gamma_{L\left(R\right)}^{k_{∥}}\left(E\right)=i\left[\sum_{L\left(R\right)}^{k_{∥}}-{\left(\sum_{L\left(R\right)}^{k_{∥}}\right)}^{†}\right]$ represents the level broadening originating from the left (right) electrode expressed in terms of electrode self-energy $\sum_{L\left(R\right)}^{k_{∥}}$[24,37]. Self-energy was calculated using an exact diagonalization of the Hamiltonian [38].

## 3. Results and Discussion

**Figure 1:**
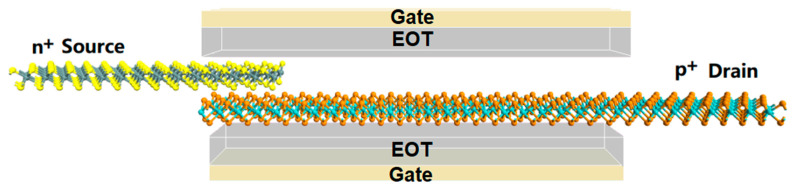
Atomic configuration of a monolayer MoTe_2_ FET with HfS_2_ located in the source region.

**Figure 2:**
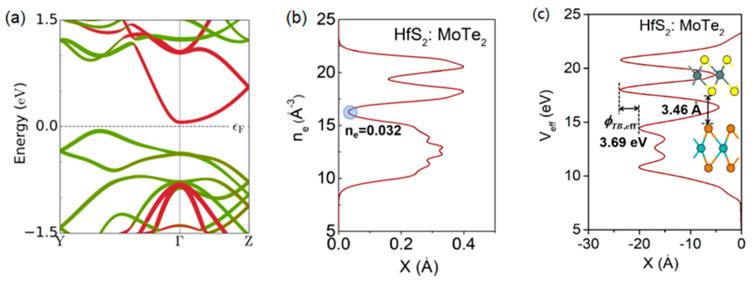
(**a**) Band structures of the HfS_2_/MoTe_2_ heterojunction. The electronic states contributed by HfS_2_ and MoTe_2_ are marked by the red and green curves, respectively. The Fermi level is referenced to zero. (**b**) Average electron density, n_e_. (**c**) Effective potential, V_eff_.

For our simulation, a transistor consisting of an intrinsic MoTe_2_ channel, an n-doped HfS_2_ source, and a p-doped MoTe_2_ drain was built, as illustrated in Figure 1. The doping concentration of the HfS_2_ was 1.78 × 10^14^ cm^−2^, and the drain region was 3.56 × 10^13^ cm^−2^. The channel length (or physical gate length) was selected to be 7.8 nm, the equivalent oxide thickness (EOT) was set to be 0.45 nm, and the corresponding dielectric constant was 3.9. Here, EOT indicates how thick a silicon oxide film would need to be to produce the same effect as the high-k material being used. The calculated lattice constant of the monolayer MoTe_2_ was a = 3.56 Å, b = 6.17 Å, and the band gap was 1.04 eV, which values are comparable to previous results [21,23]. The obtained hole mobility for the MoTe_2_ was 352.23 cm^2^·V^−1^·s^−1^, and the electron mobility was 105.13 cm^2^·V^−1^·s^−1^. Figure 2a shows the band structure of the n-doped HfS_2_ and the intrinsic MoTe_2_ heterojunction. It was observed that the MoTe_2_ was spontaneously p-type-doped by interfacing with the n-doped HfS_2_ and formed a low p-type Schottky barrier, resulting in a truncated decaying hot-carrier density with an increasing p-type channel barrier. The interfacial interaction of HfS_2_/MoTe_2_ can be described by the electron density and the effective potential, as shown in Figure 2b,c. The average electron density n_e_ perpendicular to the interface was 0.032. The effective tunnel barrier height *Φ*_TB,eff_ = 3.69 eV is defined as the minimum barrier height that an electron from the HfS_2_ has to overcome to reach the potential energy of the MoTe_2_, and the barrier width *d* = 3.46Å is the equilibrium distance between the chalcogenide atoms. The electron density and effective potential indicate that the interface interaction of HfS_2_/MoTe_2_ is not very robust, which implies a weak Fermi-level pinning effect in the HfS_2_/MoTe_2_ FET.

**Figure 3:**
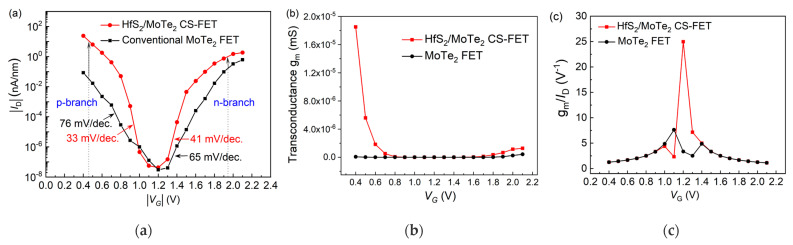
(**a**) Current-voltage transfer characteristics, (**b**) transconductance and (**c**) transconductance efficiency of MoTe_2_ FETs with and without HfS_2_ contact at different gate voltages, |*V*_SD_| = 0.74 V.

In the following, we describe the simulation of the transfer characteristics of the MoTe_2_ FET with HfS_2_ contact, as shown in Figure 3a. Interestingly, the HfS_2_/MoTe_2_ FET still showed excellent ambipolar transfer characteristics, namely, the capability of integrating p- and n-type electrical performance into a single device by utilizing identical semiconducting materials. This also indicates that the vdW interaction between the HfS_2_/MoTe_2_ interface can significantly reduce the Fermi-level pinning effect in the device. Compared to the conventional MoTe_2_ FET (with other geometric and electronic parameters kept the same), both the p- and n-type transport regions of the HfS_2_/MoTe_2_ FET are effective in reducing the SS below 60 mV/decade and significantly improving the driving currents. Specifically, the minimum SS was as low as 33 mV/decade in the p-type branch and 41 mV/decade in the n-type branch. The p-type branch shows a high on-off current ratio of 10^8^ in the *V*_G_ sweeping region from 0.46 V to 1.2 V at *V*_SD_ = 0.74 V, which is an enhancement of more than two orders of magnitude compared to the conventional case, while the low off-state current remains unaltered (*V*_G_ = 1.2 V). These results indicate that the HfS_2_ cold source can effectively optimize the performance of the conventional MoTe_2_ FET. Moreover, the other important analog performance parameters, such as transconductance (g_m_) and transconductance efficiency (g_m_/*I*_D_), are shown in Figure 3b,c. These were visualized using HfS_2_ as the source electrode, which provided a higher g_m_, resulting in a higher *I*_D_. The ratio of g_m_/I_D_ determines a transistor’s ability to regulate current efficiently. Hence, g_m_/I_D_ increases with g_m_, which in turn will increase the overall performance of the device.

**Figure 4:**
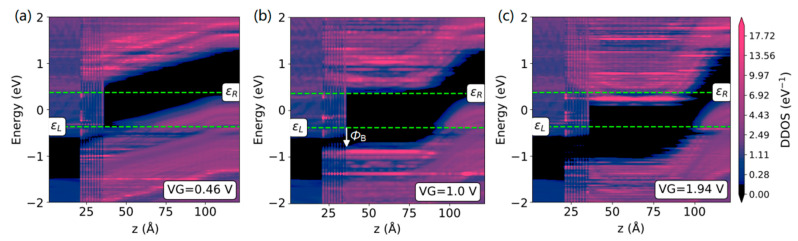

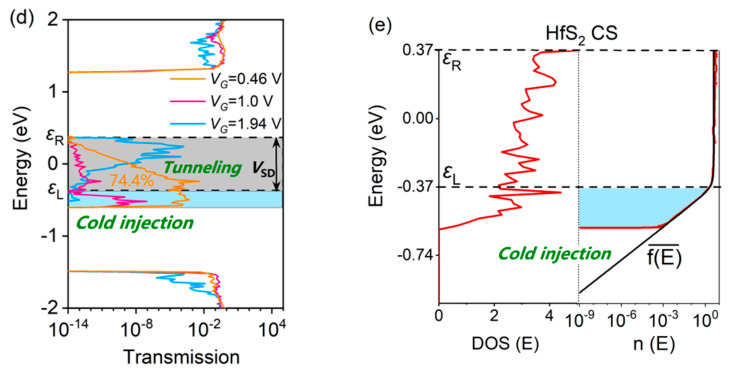
Device density of states (DDOS) projected to source, channel, and drain regions of the HfS_2_/MoTe_2_ CS-FET. DDOSs at (**a**) the on-state of the p-type transport (*V*_G_ = 0.46 V), (**b**) the steepest SS state of the p-type transport (*V*_G_ = 1.0 V), and (**c**) the on-state of the n-type transport (*V*_G_ = 1.94 V). Zero energy is set to be the average Fermi level of the source (ε_L_) and drain (ε_R_). (**d**) Comparison of the transmission spectrum at different states. (**e**) Density-of-states-energy relation *D* (*E*) and carrier density *n* (*E*) of the n-doped HfS_2_ source. *n* (*E*) shows the desired truncated decreasing (or the super-exponentially decreasing) trend. For comparison, the exponentially decreasing Boltzmann distribution function $\bar{f(E)}$ that equals *n* (*E*) at the source Fermi level ε_L_ is shown by the black curve.

To understand the transmission mechanism described above, we investigated the device density-of-states and transmission spectra at different gate voltages. For the HfS_2_/MoTe_2_ FET, it can be seen in Figure 4a,b that the height of the p-type source–channel barrier is gate-tunable, with a low barrier height for easy electron injection in the on state (*V*_G_ = 0.46 V) and a high barrier height *Φ*_B_ to block electron injection in the steepest SS state of 33 mV/decade (*V*_G_ = 1.0 V). The fact that the p-type HfS_2_/MoTe_2_ FET achieves a low SS can be explained by reference to Figure 4e. It was found that the hot-carrier density *n* (*E*) (*n* (*E*) = *DOS* (*E*) × *f* (*E*)) below the Fermi level ε_L_ showed a truncated decreasing (or super-exponentially decreasing) trend for HfS_2_ sources, rather than the exponentially decreasing trend $\bar{f(E)}$ observed for traditional metal sources. Here, the band gap of the HfS_2_ semiconductor acts as an efficient n-type cold-injection source for the p-type MoTe_2_ transistor, greatly truncating the Boltzmann hot tail and producing the steepest SS values. Both the truncated *n* (*E*) relation and the weak vdW interaction are critical to the device performance. In addition, in Figure 3, it is worth noting that the on-state current of the p-type HfS_2_/MoTe_2_ FET is higher than that of the conventional MoTe_2_ FET. As can be seen from Figure 4a, as the conduction band of the MoTe_2_ channel and the valence band of the HfS_2_ source overlapped within the bias window *V*_SD_ = ε_R_-ε_L_, an obvious source-to-drain direct tunneling occurred in the device. The dominant working mechanism at different gate voltages can be further distinguished from the transmission spectrum. It can be seen in Figure 4d that when *V*_G_ = 0.46 V, the tunneling probability of the HfS_2_/MoTe_2_ FET reached 74.4%, greatly improving the current. On the other hand, the HfS_2_/MoTe_2_ FET showed excellent ambipolar characteristics. Since the source and drain of the HfS_2_/MoTe_2_ FET are oppositely doped, for the n-type branch, the conduction band of the MoTe_2_ channel can be electrostatically tuned to the bias window with the increases in *V*_G_, as shown in Figure 4c. Thus, the carrier transport directly occurs from the conduction band to the valence band, that is, the inter-band tunneling through the band gap at the channel-drain interface, just like the tunneling FET. Consequently, a low SS of 41 mV/decade can be observed in Figure 3, and a high tunneling probability can be found in Figure 4d at *V*_G_ = 1.94 V. Therefore, a hybrid transport mechanism combined with the cold injection and the tunneling effect can effectively break through the SS limit and improve the on-state current of a conventional 2D FET.

## 4. Conclusions

Our simulation demonstrates the feasibility of using the 2D semiconductor HfS_2_ as an efficient CS to produce a steep-slope p- and n-type MoTe_2_ transistor. The truncated decreasing n(E) relation, the weak vdW interaction, and the tunneling effect are critical to the performance of MoTe_2_ FET. Specifically, the band gap of the HfS_2_ source could effectively suppress the carrier injection into the MoTe_2_ channel, resulting in a low SS of 33 mV/decade in the p-type HfS_2_/MoTe_2_ FET. The direct source-to-drain tunneling effect can increase the on-state current by more than two orders of magnitude. On the other hand, we found that in n-type HfS_2_/MoTe_2_ FET, the inter-band tunneling behavior at the channel-drain interface can reduce SS to 41 mV/decade. Therefore, such a hybrid transport mechanism in HfS_2_/MoTe_2_ FET can be used as an efficient strategy to optimize the on-state current and SS of the next-generation 2D FETs.

## Data Availability

The data that support the findings of this study are available from the corresponding author upon reasonable request.
